# Supplementary material for: A conserved cell-type gradient across the human mediodorsal and paraventricular thalamus
Source: bioRxiv. 2024 Sep 5:2024.09.03.611112. Preprint. [Version 1] doi: 10.1101/2024.09.03.611112 (PMC11398375; doi:10.1101/2024.09.03.611112)
Supplement: Supplement 2 [file NIHPP2024.09.03.611112v1-supplement-2.pdf]

bioRxiv preprint doi: https://doi.org/10.1101/2024.09.03.611112; this version posted September 5, 2024. The copyright holder for this preprint (which was not certified by peer review) is the author/funder, who has granted bioRxiv a license to display the preprint in perpetuity. It is made available under aCC-BY 4.0 International license.

Supplementary data table captions

- Supplementary data table 1 – Marker genes for human MD clusters
- Supplementary data table 2 – Marker genes for mouse MD clusters
- Supplementary data table 3 – Gene ontology enrichment analysis
- Supplementary data table 4 – MAGMA gene set test statistics
